# Renin-angiotensin system inhibitors improve the survival of cholangiocarcinoma: a propensity score-matched cohort study

**DOI:** 10.1186/s12885-023-11152-2

**Published:** 2023-09-05

**Authors:** Xiao-Xu Zhu, Jian-Hui Li, Peng Fang, Xiao-Fei Qu, Li-Jian Liang, Jia-Ming Lai, Xiao-Yu Yin

**Affiliations:** Department of Pancreato-Biliary Surgery, The First Affiliated Hospital, Sun Yat-sen University, 58 Zhongshan 2nd Rd, Guangzhou, Guangdong 510080 China

**Keywords:** Cholangiocarcinoma, Hypertension, Anti-hypertensive drugs, Angiotensin converting enzyme inhibitors, Angiotensin receptor blockers, Prognosis, Propensity score

## Abstract

**Background:**

Hypertension is a risk factor for cholangiocarcinoma (CCA). The effect of anti-hypertensive drugs on the prognosis of CCA is not clear.

**Methods:**

This is a retrospective study of 102 patients (56.9% males, median age 66 years) diagnosed with CCA and hypertension concurrently and received radical surgery (R0), with a median follow-up of 36.7 months. Kaplan-Meier analysis, Cox regressions, and propensity score (PS) matching were applied for statistical analysis.

**Results:**

Results of multivariable cox analysis showed that renin-angiotensin system inhibitors (RASis) usage was a protective factor for progression-free survival (PFS) (hazard ratio [HR] = 0.55, 95% confidence interval [95% CI]: 0.32–0.96) and overall survival (OS) (HR = 0.40, 95% CI: 0.20–0.79), respectively. Calcium channel blockers, diuretics, and β-blockers didn’t show significant associations. The association of RASis usage and PFS and OS was derived by PS matching, with a cohort of 28 RASis users and 56 RASis non-users. The median PFS and OS of RASis users (PFS, 17.6 months (9.2–34.4); OS, 24.8 months (16.5–42.3)) were longer than RASis non-users (PFS, 10.5 months (4.1–24.1); OS, 14.6 months (10.6–28.4)). The 1 year, 2 years, and 3 years’ survival rates of RASis users (89.1%, 77.0%, and 65.5%) were higher than RASis non-users (70.9%, 54.0%, and 40.0%).

**Conclusions:**

RASis usage improves the survival of patients with CCA and hypertension concurrently.

**Supplementary Information:**

The online version contains supplementary material available at 10.1186/s12885-023-11152-2.

## Background

Cholangiocarcinoma (CCA) is a highly fatal and heterogenous group of biliary malignancies arising from the biliary tree, comprising 15% of all primary liver cancers [1]. At the time of diagnosis, more than 80% of patients present with unresectable or metastatic disease. Even for a small subset of patients who are diagnosed with a localized, resectable tumor, the prognosis remains poor, with a recurrence rate of 32%, a median overall survival (OS) of 36.4 months, and a five-year survival of 25–45% after radical resection [2–5]. Combination of gemcitabine and cisplatin is the fist line adjuvant therapy for advanced CCA, but the median OS is only 11.7 months, with a five-year survival of 5–10%[6]. Strategies for adjuvant therapies should be improved from every aspect of life.

CCA is characterized by its hard consistency, due to its abundant fibrous tissues, when compared with other liver cancers. The association between CCA occurrence and cirrhosis had been confirmed by several studies [7, 8]. Angiotensin (ANG) II, the primary component of the renin-angiotensin system, was reported increasing in patients with cirrhosis and rats with active liver fibrogenesis [9–12]. ANG II also plays an integral role in the pathogenesis of hypertension. Renin-angiotensin system inhibitors (RASis), including angiotensin converting enzyme inhibitors (ACEis) and angiotensin receptor blockers (ARBs), are generally applied for anti-hypertension. RASis were also reported to attenuate the fibrosis and improve the prognosis of various cancers [13, 14], but not well studied in CCA. In addition, hypertension is the most common comorbidity in CCA patients receiving adjuvant therapies (22.0-73.1%) [15–20]. But up to now, there is still no official guidelines for the usage strategies of anti-hypertensive drugs for patients with CCA and hypertension concurrently.

Herein, we conducted a retrospectively study to evaluate whether the use of RASis could improve the prognosis of CCA patients with hypertension.

## Methods

### Selection of patients

Patients who were histologically diagnosed with cholangiocarcinoma (CCA) and hypertension concurrently and received surgery from March 2017 to February 2022 in The First Affiliated Hospital of Sun Yat-sen University (FAHSYSU) were included retrospectively. The diagnosis of CCA was reviewed by three pathologists, including intrahepatic cholangiocarcinoma (iCCA), perihilar cholangiocarcinoma (pCCA) and distal cholangiocarcinoma (dCCA), according to the International Classification of Diseases 10 (ICD-10). Hypertension was diagnosed by patients’ medical history and patients’ systolic blood pressure (SBP, SBP ≥ 140mmHg) and/or diastolic blood pressure (DBP, DBP ≥ 90mmHg) measured before surgery. Patients’ demographics, blood pressures, laboratory parameters, surgical procedures, histological diagnosis and drug prescription and dispensing history were collected from Hospital’s Information System of FAHSYSU. Follow up results were collected by outpatient clinic visits and telephone follow up.

**Fig. 1:**
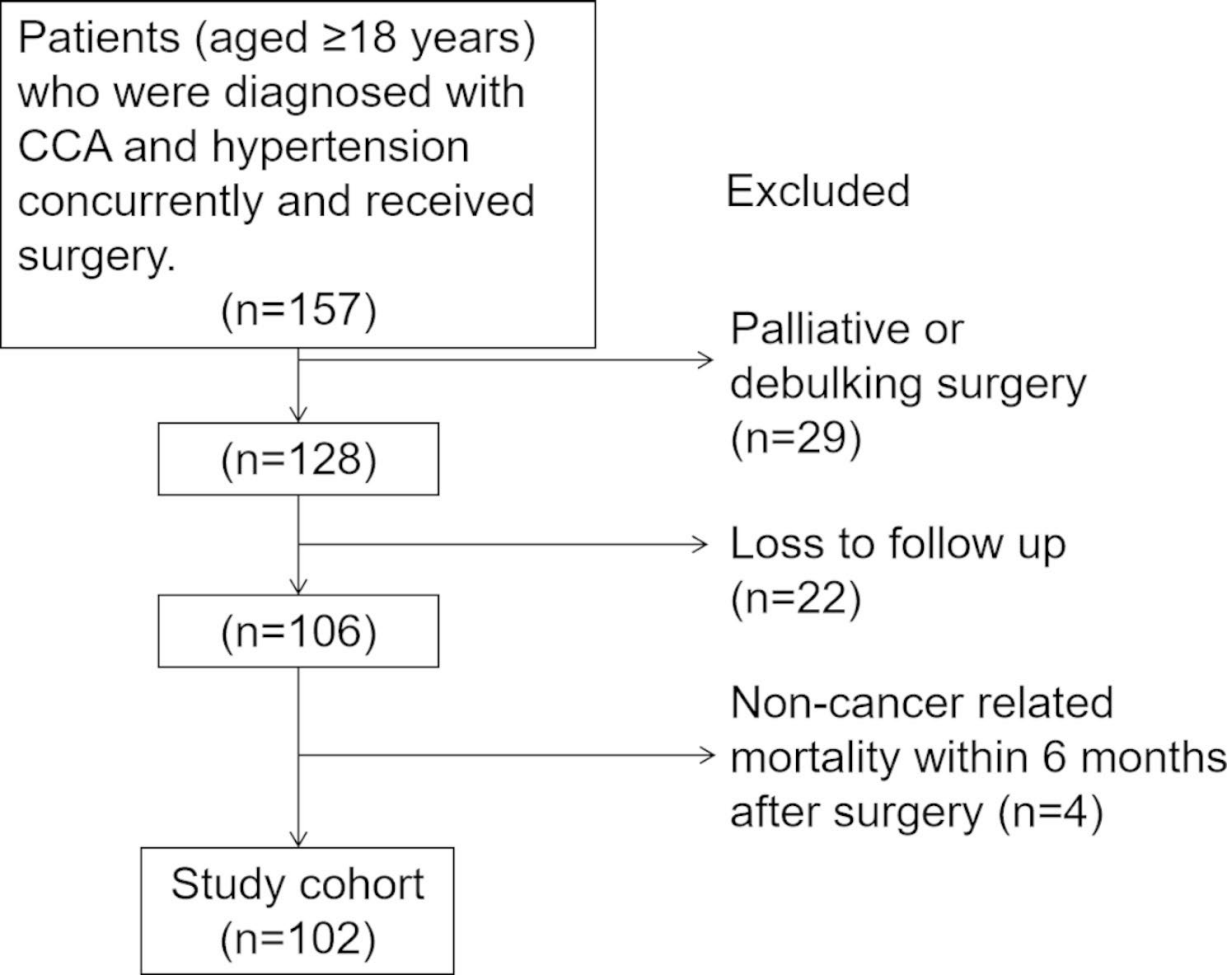
Patient selection flow diagram CCA, cholangiocarcinoma

Figure 1 illustrated the process of patients selection. 157 patients (aged ≥ 18 years) were diagnosed with CCA and hypertension concurrently and received surgery from March 2017 to February 2022 in FAHSYSU. 29 patients received palliative or debulking surgery were excluded, and only patients received R0 resection could be included. 22 patients lost to follow up were excluded. 4 patients with non-cancer related mortality within 6 months after surgery were also excluded. Finally, 102 patients were enrolled in the study cohort.

### Outcome definition

The primary outcome of interest was overall survival (OS),defined as the survival from surgical operation to cancer-related mortality. The secondary outcome was progression-free survival (PFS), defined as the survival from surgical operation to tumor recurrence or metastasis detected by imaging. We followed up patients till August 31, 2022.

### Study variables

The exposure of interest was the administration of anti-hypertensive drugs, defined as any anti-hypertensive drugs taken at least 6 months continuously from 1 year before surgery to the outcome time or the last follow up time point. Four classes of anti-hypertensive drugs were included as follows: (1) renin-angiotensin system inhibitors (RASis), (2) calcium channel blockers (CCBs), (3) Diuretics, (4) β-blockers.

Other covariates included patients’ characteristics, blood pressures, tumor characteristics, laboratory parameters and adjuvant therapies. Patients’ characteristics were sex, age of receiving surgeries and comorbidity (biliary stone). Blood pressures were measured in the morning of the second day after patients admitted to hospital. Tumor characteristics included histological diagnosis, tumor size, tunor differentiation, perineural invasion, vascular cancer embolus, adjacent organ invasion, intrahepatic metastasis and lymph node metastasis. Laboratory parameters included TBIL, DBIL, IBIL, ALB, ALBI score/grade, urea, creatinine, serum potassium, CA19-9, CEA and CA125. Adjuvant therapies were just classified as yes or not, regardless of the therapeutic regimens.

### Statistical analysis

Continuous variables were expressed as median and interquartile range (IQR). Categorical variables were expressed as percentages. Continuous variables were compared with Mann-Whitney U test. Categorical variables were compared with χ2 test or Fisher exact test as appropriate. Survival outcomes (OS and PFS) were assessed by the Kaplan-Meier method. The survival rates were compared by log-rank test. Cox regressions were expressed as hazard ratio [HR] with confidence interval [95% CI]. Prognostic factors associated with OS and PFS were assessed using univariable and multivariable Cox regressions. Variables, in univariable Cox regressions, with p < 0.200 were included in the multivariable Cox regression.

When compared the effect of anti-hypertensive drugs on outcome, propensity scores (PS) matching was used to control for confounding due to potential selection bias in the cohort study. The PS was estimated by multivariable logistic regression based on the variables included in this study. RASis users were matched to non-users in a 1:3 ratio without replacement using a greedy distance-based matching algorithm with the logit of the PS within 0.2 standard deviation. After PS matching, balance was evaluated using absolute standardized differences (ASD) for all covariates. An ASD of < 0.20 was considered an good balance.

All analyses were performed by IBM SPSS statistics 22.0 with PS matching plug-in. A two-tailed p < 0.05 was considered statistically significant for all tests unless indicated otherwise.

## Results

### Characteristics of patients with CCA

A total of 102 patients diagnosed with CCA and hypertension concurrently and received radical surgery were enrolled in this study. The main characteristics of the population were summarized in Table 1. There were 58 (56.9%) males in this cohort with a median age of 66.0 years (IQR: 58.0–72.0). Each patient’s blood pressure of the second morning after admission was collected, the median systolic blood pressure (SBP) and diastolic blood pressure (DBP) were 138 mmHg (IQR: 127–146) and 81 mmHg (IQR: 74–90) respectively. With respect to use of anti-hypertensive drugs, 39 (38.2%) patients took RASis, 69 (67.6%) took CCBs, 7 (6.9%) took diuretics, 20 (19.6%) took β-blockers. In terms of tumor features, there were 68 (66.7%) iCCA, 24 (23.5%) pCCA and 10 (9.8%) dCCA with the median tumor size of 55.0 mm (45.0-75.5), 20.0 mm (18.0–30.0), and 16.5 mm (14.0–22.0), respectively. Based on results of imaging and histology, 5 (4.9%) tumors were low differentiated, 93 (91.2%) tumors were moderate differentiated, 4 (3.9%) tumors were high differentiated, 37 (36.3%) had perineural invasion, 10 (9.8%) had vascular cancer embolus, 5 (4.9%) had adjacent organ invasion, 8 (7.8%) had intrahepatic metastasis, 25 (24.5%) had lymph node metastasis and 35 (34.3%) with biliary stone. 22 (21.6%) patients received adjuvant therapy. The median ALBI score was − 2.47 (IQR: -2.73–1.97) with 36 (35.3%), 56 (54.9%), and 10 (9.8%) of ALBI grade 1, 2, and 3, respectively. The median values of urea, creatinine, and serum potassium were 5.0 (4.2–6.5) mmol/L, 72 (59–82) µmol/L, and 3.98 (3.71–4.29) mmol/L, respectively. The median values of CA19-9, CEA and CA125 were 124.8 U/L (18.1-1116.9), 3.0 ng/ml (2.1–6.3), and 17.7 U/L (10.7–35.8), respectively.

**Table 1:** Characteristics of patients with CCA

Characteristics	n = 102
Sex, Male, n (%)	58 (56.9%)
Age (years)	66.0 (58.0–72.0)
Anti-hypertensive drugs, n (%)	
RASis	39 (38.2%)
CCBs	69 (67.6%)
Diuretics	7 (6.9%)
β-blockers	20 (19.6%)
Blood pressure	
SBP (mmHg)	138 (127–146)
DBP (mmHg)	81 (74–90)
Diagnosis, n (%)	
iCCA	68 (66.7%)
pCCA	24 (23.5%)
dCCA	10 (9.8%)
Tumor size (mm)	
iCCA	55.0 (45.0-75.5)
pCCA	20.0 (18.0–30.0)
dCCA	16.5 (14.0–22.0)
Tumor differentiation	
low	5 (4.9%)
moderate	93 (91.2%)
high	4 (3.9%)
Perineural invasion, n (%)	37 (36.3%)
Vascular cancer embolus, n (%)	10 (9.8%)
Adjacent organ invasion, n (%)	5 (4.9%)
Intrahepatic metastasis, n (%)	8 (7.8%)
Lymph node metastasis, n (%)	25 (24.5%)
Biliary stone, n (%)	35 (34.3%)
TBIL (µmol/L)	17.4 (11.7–67.6)
DBIL (µmol/L)	3.7 (2.2–56.0)
IBIL (µmol/L)	13.3 (9.3–42.7)
ALB (g/L)	38.9 (36.2–41.4)
ALBI score	-2.47 (-2.73–1.97)
ALBI grade	
1	36 (35.3%)
2	56 (54.9%)
3	10 (9.8%)
UREA (mmol/L)	5.0 (4.2–6.5)
CREA (µmol/L)	72 (59–82)
K^+^ (mmol/L)	3.98 (3.71–4.29)
CA19-9 (U/L)	124.8 (18.1-1116.9)
CEA (ng/ml)	3.0 (2.1–6.3)
CA125 (U/L)	17.7 (10.7–35.8)
Adjuvant therapy, n (%)	22 (21.6%)

Continuous variables were expressed as median with interquartile range

Categorical variables were expressed as number (%)

Abbreviations:

RASi, renin-angiotensin system inhibitor; CCB, calcium channel blocker; SBP, systolic blood pressure; DBP, diastolic blood pressure; iCCA, intrahepatic cholangiocarcinoma; pCCA, perihilar cholangiocarcinoma, dCCA, distal cholangiocarcinoma; TBIL, total bilirubin; DBIL, direct bilirubin; IBIL, indirect bilirubin; ALB, albumin; ALBI, albumin-bilirubin; CREA, creatinine; K^+^, serum potassium; CA19-9, carbohydrate antigen 19 − 9; CEA, carcinoembryonic antigen; CA125, cancer antigen 125

### Outcomes of patients

The median follow-up of the 102 patients was 36.7 months, with a median PFS of 12.4 months (6.2–24.5) and a median OS of 20.1 months (11.5–35.2). The 1 year, 2 years, and 3 years’ survival rates were 79.9%, 60.2%, and 49.4%, respectively. Results of univariable and multivariable cox analysis showed that RASis use (HR = 0.55, 95% CI: 0.32–0.96, p = 0.034), perineural invasion (HR = 2.09, 95% CI: 1.21–3.62, p = 0.009), and lymph node metastasis (HR = 1.80, 95% CI: 1.00-3.24, p = 0.050) were independently associated with PFS. RASis use (HR = 0.40, 95% CI: 0.20–0.79, p = 0.008), perineural invasion (HR = 2.25, 95% CI: 1.21–4.19, p = 0.011), and lymph node metastasis (HR = 2.10, 95% CI: 1.10–3.99, p = 0.024) were independently associated with OS (Tables 2 and 3). Kaplan-Meier curves of PFS and OS according to RASis use, perineural invasion, and lymph node metastasis were shown in **Supplementary Fig. **1. These results indicated that RASis use was a protective factor for the survival of CCA.

**Table 2:** Univariable analyses of factors associated with PFS and OS

Univariable cox regression						
	PFS			OS		
	HR	95% CI	*P*	HR	95% CI	*P*
Sex, Male	0.81	0.49–1.35	0.423	0.89	0.49–1.60	0.692
Age, ≥ 67 years	1.04	0.63–1.75	0.866	1.43	0.79–2.57	0.234
Anti-hypertensive drugs						
RASis	0.575	0.33–0.99	0.047	0.39	0.20–0.76	0.006
CCBs	1.00	0.58–1.71	0.999	0.91	0.50–1.66	0.760
Diuretics	0.18	0.03–1.29	0.088	0.29	0.04–2.10	0.220
β-blockers	0.82	0.43–1.59	0.561	1.01	0.50–2.04	0.974
SBP, ≥ 140mmHg	1.19	0.71–1.98	0.515	1.25	0.70–2.23	0.452
DBP, ≥ 90mmHg	1.18	0.71–1.97	0.526	1.24	0.70–2.21	0.465
Diagnosis						
iCCA	Ref			Ref		
pCCA	1.11	0.61–2.03	0.727	1.27	0.65–2.45	0.484
dCCA	1.21	0.54–2.73	0.638	1.23	0.47–3.19	0.674
Tumor size*	1.48	0.83–2.63	0.183	1.79	0.91–3.54	0.094
Tumor differentiation						
low	Ref			Ref		
moderate	3.83	0.40-36.89	0.971	4.19	0.43–40.61	0.568
high	4.16	0.57–30.18	0.229	3.12	0.43–22.82	0.581
Perineural invasion	2.28	1.35–3.83	0.002	2.58	1.42–4.69	0.002
Vascular cancer embolus	2.43	1.15–5.16	0.020	1.30	0.51–3.31	0.579
Adjacent organ invasion	1.82	0.66–5.04	0.249	1.76	0.54–5.70	0.346
Intrahepatic metastasis	1.83	0.77–4.20	0.172	1.58	0.62–4.01	0.337
Lymph node metastasis	2.39	1.37–4.18	0.002	2.96	1.59–5.51	0.001
Biliary stone	0.85	0.49–1.47	0.565	0.92	0.50–1.70	0.788
TBIL, > 22µmol/L	0.81	0.48–1.38	0.441	0.69	0.37–1.30	0.247
DBIL, > 7µmol/L	0.77	0.45–1.32	0.344	0.71	0.38–1.33	0.285
IBIL, > 15µmol/L	0.85	0.50–1.42	0.524	0.78	0.43–1.41	0.414
ABL, < 35 g/L	1.04	0.56–1.93	0.897	1.00	0.50–2.03	0.991
ALBI						
1	Ref			Ref		
2	1.48	0.83–2.66	0.184	1.33	0.68–2.57	0.404
3	1.70	0.71–4.11	0.237	1.84	0.74–4.60	0.190
UREA, > 8.6mmol/L	3.75	0.85–16.64	0.082	2.44	0.55–10.89	0.244
CREA, > 115µmol/L	0.57	0.19–1.66	0.299	0.62	0.18–2.13	0.444
CA19-9, > 35U/L	1.75	0.98–3.11	0.059	2.06	1.04–4.07	0.038
CEA, >5ng/ml	1.42	0.83–2.44	0.204	1.12	0.58–2.10	0.725
CA125, > 35U/L	0.95	0.53–1.71	0.859	0.87	0.44–1.72	0.682
Adjuvant therapy				2.41	1.25–4.62	0.008

Abbreviations: PFS, progression-free survival; OS, overall survival; HR, hazard ratio; CI, confidence interval; ASD, absolute standardized difference; RASi, renin-angiotensin system inhibitor; CCB, calcium channel blocker; SBP, systolic blood pressure; DBP, diastolic blood pressure; iCCA, intrahepatic cholangiocarcinoma; pCCA, perihilar cholangiocarcinoma, dCCA, distal cholangiocarcinoma; TBIL, total bilirubin; DBIL, direct bilirubin; IBIL, indirect bilirubin; ALB, albumin; ALBI, albumin-bilirubin; CREA, creatinine; CA19-9, carbohydrate antigen 19 − 9; CEA, carcinoembryonic antigen; CA125, cancer antigen 125

* Tumor size: iCCA, tumor diameter > 5 cm; pCCA, tumor invade beyond ductal wall; dCCA, depth of tumor infiltration > 5 mm

**Table 3:** Multivariable analyses of factors associated with PFS and OS

Multivariable cox regression**						
	PFS			OS		
	HR	95% CI	*P*	HR	95% CI	*P*
RASis	0.55	0.32–0.96	0.034	0.40	0.20–0.79	0.008
Perineural invasion	2.09	1.21–3.62	0.009	2.25	1.21–4.19	0.011
Lymph node metastasis	1.80	1.00-3.24	0.050	2.10	1.10–3.99	0.024
Tumor size*			0.201			0.052
Vascular cancer embolus			0.127			
Intrahepatic metastasis			0.124			
Diuretics			0.063			
CA19-9, > 35U/L			0.126			0.081
ALBI grade						
1			Ref			Ref
2			0.316			0.446
3			0.603			0.936
Adjuvant therapy						0.088

Abbreviations: PFS, progression-free survival; OS, overall survival; HR, hazard ratio; CI, confidence interval; ASD, absolute standardized difference; RASi, renin-angiotensin system inhibitor; CCB, calcium channel blocker; SBP, systolic blood pressure; DBP, diastolic blood pressure; iCCA, intrahepatic cholangiocarcinoma; pCCA, perihilar cholangiocarcinoma, dCCA, distal cholangiocarcinoma; TBIL, total bilirubin; DBIL, direct bilirubin; IBIL, indirect bilirubin; ALB, albumin; ALBI, albumin-bilirubin; CA19-9, carbohydrate antigen 19 − 9; CEA, carcinoembryonic antigen; CA125, cancer antigen 125

* Tumor size: iCCA, tumor diameter > 5 cm; pCCA, tumor invade beyond ductal wall; dCCA, depth of tumor infiltration > 5 mm

** Variables included in the multivariable Cox model were selected based on the results of the univariable analysis (i.e., all variables associated to OS with a p value < 0.200)

### Association between patients’ survival and RASis use

Anti-hypertensive drugs were classified to four groups based on their pharmacological mechanism, including RASis, CCBs, diuretics, and β-blockers. **Supplementary Table **1 showed the detailed anti-hypertensive drugs.

According to the results of univariable and multivariable cox analysis, RASis use was associated with the survival (PFS and OS) of CCA patients. It’s a very interesting result and was not reported before. To verify this result, we performed PS matching to balance the bias between RASis users and non-users. The baseline characteristics were shown in **Supplementary Table **2. There were a total of 102 patients, including 39 RASis users and 63 RASis non-users. Before PS matching, some covariates between RASis users and non-users were imbalanced (ASD > 0.20), including sex (male, ADS = 0.241), diuretics use (ASD = 0.377), β-blockers use (ASD = 0.305), DBP (ASD = -0.215), diagnosis (iCCA, ASD = -0.250; dCCA, ASD = 0.339), tumor size (ASD = 0.210), lymph node metastasis (ASD = -0.273), and adjuvant therapy (ASD = -0.274). After PS matching, 84 patients were enrolled in the cohort, including 28 RASis users and 56 RASis non-users. All covariates were well balanced (ASD < 0.20). The characteristics of the PS-matched cohort were shown in Table 4.

**Table 4:** Characteristics of the study cohort after PS matching

	All (n = 84)	RASis users (n = 28)	RASis non-users (n = 56)	ASD*
Sex, Male, n (%)	43 (51.2%)	14 (50%)	29 (51.8%)	-0.035
Age (years)	66.0 (57.5–72.0)	67.5 (56.5–72.0)	66.0 (58.0–72.0)	-0.020
Anti-hypertensive drugs, n (%)				
CCBs	57 (67.9%)	20 (71.4%)	37 (66.1%)	-0.116
Diuretics	2 (2.4%)	1 (3.6%)	1 (1.8%)	0.094
β-blockers	13 (15.5%)	5 (17.9%)	8 (14.3%)	0.092
Blood pressure (mmHg)				
SBP	138 (127–147)	139 (124–149)	137 (127–146)	-0.068
DBP	82 (76–90)	78 (75–89)	82 (76–91)	-0.140
Diagnosis, n (%)				
iCCA	61 (72.6%)	20 (71.4%)	41 (73.2%)	-0.040
pCCA	19 (22.6%)	6 (21.4%)	13 (23.2%)	-0.043
dCCA	4 (4.8%)	2 (7.1%)	2 (3.6)	0.136
Tumor size (mm)	46.0 (28.0-62.5)	44.5 (20.5–62.5)	50.0 (30.0-62.5)	-0.052
Perineural invasion, n (%)	29 (34.5%)	11 (39.3%)	18 (32.1%)	0.144
Vascular cancer embolus, n (%)	7 (8.3%)	2 (7.1%)	5 (8.9%)	-0.068
Adjacent organ invasion, n (%)	2 (2.4%)	0 (0.0%)	2 (3.6%)	0.000
Intrahepatic metastasis, n (%)	8 (9.5%)	3 (10.7%)	5 (8.9%)	0.057
Lymph node metastasis, n (%)	16 (19.0%)	5 (17.9%)	11 (19.6%)	-0.046
Biliary stone, n (%)	28 (33.3%)	9 (32.1%)	19 (33.9%)	0.038
TBIL (µmol/L)	16.8 (11.5–52.4)	17.9 (12.2–67.0)	16.2 (10.8–43.5)	0.138
DBIL (µmol/L)	3.2 (2.1–29.7)	3.4 (2.3–28.4)	3.2 (2.1–29.7)	0.009
IBIL (µmol/L)	13.1 (9.2–30.7)	14.1 (9.7–38.6)	12.6 (8.8–24.4)	0.138
ALB (g/L)	38.9 (36.3–41.6)	38.4 (36.8–41.7)	39.5 (35.9–41.5)	-0.011
ALBI score	-2.51 (-2.76–1.99)	-2.48 (-2.74–2.20)	-2.54 (-2.77–1.91)	-0.166
CA19-9 (U/L)	206.6 (21.0-1058.5)	212.5 (21.0-1350.9)	171.3 (18.9-797.7)	0.138
CEA (ng/ml)	2.9 (2.1–7.2)	3.0 (2.3–8.1)	2.9 (1.9–6.1)	0.073
CA125 (U/L)	17.6 (10.6–33.7)	19.9 (9.9–35.8)	17.3 (11.1–33.7)	0.173
Adjuvant therapy, n (%)	20 (23.8%)	6 (21.4%)	14 (25.0%)	-0.085

Continuous variables were expressed as median with interquartile range

Categorical variables were expressed as number (%)

Abbreviations: PS, propensity score; ASD, absolute standardized difference; RASi, renin-angiotensin system inhibitor; CCB, calcium channel blocker; SBP, systolic blood pressure; DBP, diastolic blood pressure; iCCA, intrahepatic cholangiocarcinoma; pCCA, perihilar cholangiocarcinoma, dCCA, distal cholangiocarcinoma; TBIL, total bilirubin; DBIL, direct bilirubin; IBIL, indirect bilirubin; ALB, albumin; ALBI, albumin-bilirubin; CA19-9, carbohydrate antigen 19 − 9; CEA, carcinoembryonic antigen; CA125, cancer antigen 125

^*^ Variables with an ASD > 0.20 is considered to be not well balanced

After PS matching, the median PFS and OS of RASis users (PFS, 17.6 months (9.2–34.4); OS, 24.8 months (16.5–42.3)) were longer than RASis non-users (PFS, 10.5 months (4.1–24.1); OS, 14.6 months (10.6–28.4)). RASis use was associated with a lower risk of PFS (HR = 0.72, 95% CI: 0.39–1.32, p = 0.283) and OS (HR = 0.42, 95% CI: 0.19–0.91, p = 0.028). Kaplan-Meier curves of PFS and OS according to RASis use were shown in **Supplementary Fig. **2. The 1 year, 2 years, and 3 years’ survival rates of RASis users (89.1%, 77.0%, and 65.5%) were higher than RASis non-users (70.9%, 54.0%, and 40.0%).

As RASis could cause azotemia and hyperkalemia, we compared the level of urea, creatinine, and serum potassium between RASis users and non-users. Table 1 and Supplementary Table 3 showed the median values of urea, creatinine, and serum potassium of the entire cohort, RASis users, and RASis non-users, respectively. Results showed that even though RASis users had higher level of urea (5.7 vs. 4.7, mmol/L) and creatinine (76 vs. 69, mmol/L) than non-users (Supplementary Table 3), the values were still in normal range. Then we compared the proportion of patients with azotemia (urea and creatinine exceeded the normal range). There were no significant difference between RASis users and non-users (Supplementary Table 5). The level of serum potassium was not significantly different between RASis users and non-users (Supplementary Table 3) and only one RASis non-user patients in this study cohort got hyperkalemia (Supplementary Table 5). We also compared the level of urea, creatinine, and serum potassium in the cohort after PS matching and similar results were obtained (Supplementary Tables 4, 6). To clarify the correlation between azotemia and survival, we did cox analysis. Results of univariable cox analysis showed that urea and creatinine were not associated with PFS and OS (Table 2).

## Discussion

As CCA commonly occurs aound 60 years old, there is a large proportion of patients are complicated with hypertension. But the anti-hypertensive guideline for patients with hypertension and CCA concurrently is blank. Up to now, the effect of anti-hypertensive drugs on the prognosis of CCA is still unclear. In this study, we described a retrospective cohort of 102 CCA patients with hypertension and CCA concurrently and received radical surgery. We have shown that RASis usage was independently associated with better PFS (HR = 0.55, 95% CI: 0.32–0.96, p = 0.034) and OS (HR = 0.40, 95% CI: 0.20–0.79, p = 0.008), but other types of anti-hypertensive drugs in this cohort, including CCBs, β-blockers, and diuretics, didn’t show the similar effect. After balancing other factors that could influence the PFS and OS by PS matching, 84 CCA patients were in the final cohort. Results shown that RASis usage improved the prognosis of patients with hypertension and CCA concurrently, with longer PFS (17.6 m vs. 10.5 m), OS (24.8 m vs. 14.6 m), and a higher 3 years’ survival rate (65.5% vs. 40.0%). These findings haven’t been reported before.

With the increase of aging population, the association between hypertension and CCA has been wildly studied. A hospital-based case-control study of 303 CCA patients, including 136 intrahepatic cholangiocarcinoma and 167 extrahepatic cholangiocarcinoma, showed that hypertension harbored strong association with CCA [21]. Other studies also proved that management of hypertension could improve the prognosis of CCA [22, 23]. These results indicated that hypertension is a risk factor for the survival of CCA. The renin-angiotensin system plays an important role in the regulation of blood pressure. Angiotensin-converting znzyme 2 (ACE2), a key component of the renin-angiotensin system, was reported wildly expressed in the biliary system [24] and increased in CCA [25, 26]. Angiotensin II (ANG II) was shown to facilitate fibrosis and tumor progression of CCA through an interaction with hepatic stellate cells [11, 12]. These researches proved that renin-angiotensin system was activated in CCA and could promote the progression of CCA. In addition, hypertension is also one of the common adverse events during the adjuvant therapy of CCA (22.0-57.7%) [17–20]. An open-label phase II prospective study of apatinib treatment for advanced CCA reported a 57.7% occurrence of hypertension [18], and hypertension was reported as the most common comorbidity in a radiation therapy of Yttrium-90 resin microspheres [20]. But up to now, there was no recommend of anti-hypertensive drugs for patients received adjuvant therapy. This is also a good point for further research.

The effect of anti-hypertensive drugs on malignancies is controversial. A total participants of 14,392 patients cohort showed that anti-hypertensive drugs were associated with lower all-cause mortality (hazard ratio [HR], 0.32; 95% CI, 0.25–0.42), cardiovascular mortality (HR, 0.33; 95% CI, 0.21–0.53), and cancer mortality (HR, 0.30; 95% CI, 0.19–0.47) [27]. A prospective cohort study of 3012 patients with gastric carcinoma undergoing radical gastrectomy showed that anti-hypertensive drugs were related to 42% (HR 0.58, 95% CI 0.47–0.73) reduced mortality risk relative to those without medications [28]. A large cohort of 90,708 lung cancer research demonstrated that the lower risk of lung cancer persisted with a longer follow-up period of anti-hypertensives usage [29]. Other studies on breast cancer [30, 31], pancreatic cancer [32], and colorectal cancer [33] also suggested that anti-hypertensive drugs could reduce the side effects of cancer treatment, and stop the reoccurrence of cancers in the survivors. But other researches have the opposite conclusion. A large-scale cohort study in Japan suggested that long-term use of antihypertensive drugs may be associated with an increased incidence of colorectal and renal cancer [34]. A population based study of 302,634 users of anti-hypertensive drugs and 605,268 non-users indicated that higher cumulative exposure to thiazides was associated with increased rates of incident skin cancer in people aged 66 years and older [35]. Other studies suggested that anti-hypertensive drugs increased the risk of kidney cancer [36] and squamous cell carcinoma [37]. These differences may due to the different types of cancers and population that enrolled in the studies. Up to now, the effect of anti-hypertensive drugs on the prognosis of CCA was not clarified.

As the renin-angiotensin system plays a key role in regulating the blood pressure. The effect of inhibitors of renin-angiotensin system on malignancies attracts lots of attention. A meta-analysis of the association between anti-hypertensive drugs use and breast cancer showed that only RASis were associated with a significantly lower breast cancer risk. β-blockers, CCBs and diuretics increased the risk of breast cancer [31]. A nationwide cohort study of 70,549 individuals from Korea showed that ARBs use was independently associated with a decreased risk of cancer overall compared to other antihypertensive drugs [38]. A Nationwide Cohort Study of 12,122 women identified from the Finnish Cancer Registry with ovarian cancer reported that ACEis confered survival benefits in women with ovarian cancer [39]. Other studies on breast cancer, colorectal cancer, lung cancer, stomach cancer, ovarian cancer, and melanoma also suggested that ACEis and/or ARBs could improve the survival [40–44].

With respect to the effect of renin-angiotensin system inhibitors on CCA, a study showed that ARBs attenuated CCA cell growth by inhibiting the oncogenic activity of Yes-associated protein [45]. Another study reported that telmisartan inhibited cell proliferation and tumor growth of CCA through cell cycle arrest [46]. But few studies on the effect of renin-angiotensin system inhibitors on CCA have been reported yet. This may due to a lower incidence of CCA compared with other cancers, including breast cancer and gastrointestinal cancer. Several studies explored the impact of RASis on patients with advanced, or recurrent, and/or metastatic CCA but got negative results [47, 48]. Herein, we described a PS matching cohort of 84 CCA patients and demonstrated that RASis usage could improve the survival of CCA patients. The possible reason was that the population of patients included in the present study was quite different from that in the previous studies. We included patients undergone radical surgery, while the previous studies included patients with unresectable tumors, which would be more difficult for RASis to yield effects.

Severe limitations of the current study should be acknowledged. First, this is not a prospective study, hence selection bias unavoidably existed and might influence the results. Secondly, this is single center study and the study cohort is not large enough, therefore large-scale prospective multicenter studies, ideally randomized controlled trials, are still warranted to verify the conclusion.

## Conclusions

In this PS matching cohort study, we have demonstrated that RASis usage improved the survival of patients with CCA and hypertension concurrently.

### Electronic supplementary material

Below is the link to the electronic supplementary material.

**Supplementary Figure 1**. Kaplan-Meier curves of progression-free survival (A) and overall survival (B) according to RASis use, perineural invasion, and lymph node metastasis. RASis, renin-angiotensin system inhibitors. **Supplementary Figure 2**. Kaplan-Meier curves of progression-free survival (A) and overall survival (B) according to RASis use after propensity score matching. RASis, renin-angiotensin system inhibitors. **Supplementary Tables: Supplementary Table 1**. Anti-Hypertensive drugs. **Supplementary Table 2**. Characteristics of the study cohort before PS matching. **Supplementary Table 3**. Supplementary characteristics of patients in the whole study cohort. **Supplementary Table 4**. Supplementary characteristics of patients in the cohort after PS matching. **Supplementary Table 5**. The proportion of azotemia and hyperkalemia of the whole study cohort. **Supplementary Table 6**. The proportion of azotemia and hyperkalemia of the cohort after PS matching

## Data Availability

The datasets used and/or analysed during the current study available from the corresponding author on reasonable request.
