# Supplementary material for: Renin-angiotensin system inhibitors improve the survival of cholangiocarcinoma: a propensity score-matched cohort study
Source: BMC Cancer. 2023 Sep 5;23:826. doi: 10.1186/s12885-023-11152-2 (PMC10478452; doi:10.1186/s12885-023-11152-2)
Supplement: Supplementary file 1 — Supplementary Figure 1. Kaplan-Meier curves of progression-free survival (A) and overall survival (B) according to RASis use, perineural invasion, and lymph node metastasis. RASis, renin-angiotensin system inhibitors. Supplementary Figure 2. Kaplan-Meier curves of progression-free survival (A) and overall survival (B) according to RASis use after propensity score matching. RASis, renin-angiotensin system inhibitors. Supplementary Tables: Supplementary Table 1. Anti-Hypertensive drugs. Supplementary Table 2. Characteristics of the study cohort before PS matching. Supplementary Table 3. Supplementary characteristics of patients in the whole study cohort. Supplementary Table 4. Supplementary characteristics of patients in the cohort after PS matching. Supplementary Table 5. The proportion of azotemia and hyperkalemia of the whole study cohort. Supplementary Table 6. The proportion of azotemia and hyperkalemia of the cohort after PS matching [file 12885_2023_11152_MOESM1_ESM.docx]

Renin-Angiotensin System Inhibitors Improve the Survival of Cholangiocarcinoma: A Propensity Score-matched Cohort Study

Xiao-Xu Zhu^1#^, Jian-Hui Li^1#^, Peng Fang^1^, Xiao-Fei Qu^1^, Li-Jian Liang^1^, Jia-Ming Lai^1*^, Xiao-Yu Yin^1*^

^1^Department of Pancreato-Biliary Surgery, The First Affiliated Hospital, Sun Yat-sen University, Guangzhou, Guangdong, China

# These authors contributed equally to this work and share first authorship.

**Supplementary Figures**


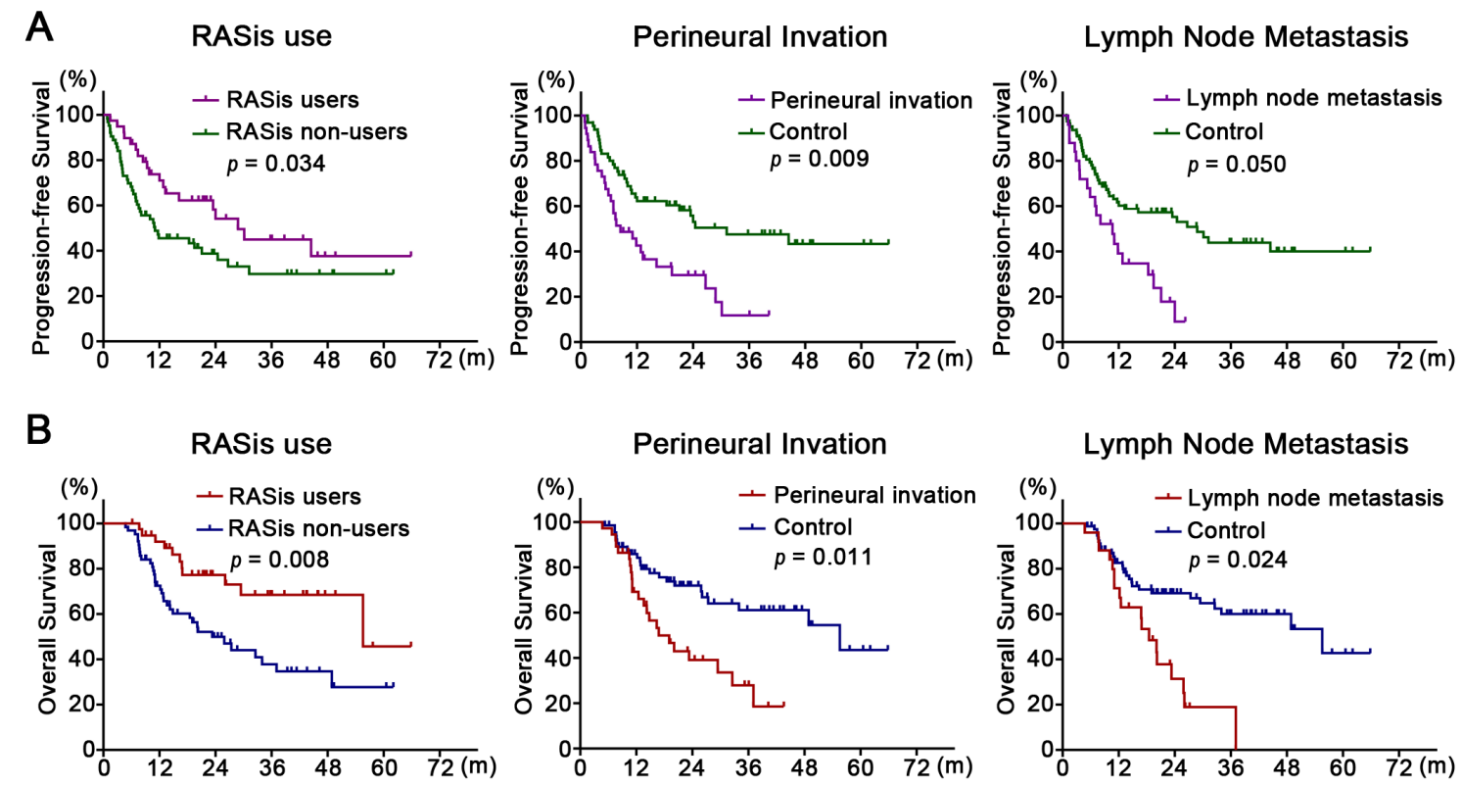


**Supplementary Figure 1.** Kaplan-Meier curves of progression-free survival **(A)** and overall survival **(B)** according to RASis use, perineural invasion, and lymph node metastasis. RASis, renin-angiotensin system inhibitors.


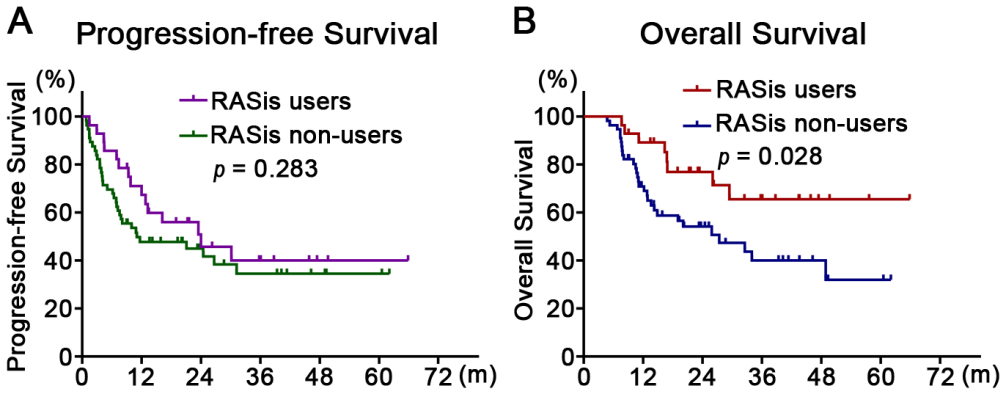


**Supplementary Figure 2.** Kaplan-Meier curves of progression-free survival **(A)** and overall survival **(B)** according to RASis use after propensity score matching. RASis, renin-angiotensin system inhibitors.

## Supplementary Tables

**Supplementary Table 1. Anti-Hypertensive drugs**

| **Anti-Hypertensive Drugs** | **Patient numbers** |
| --- | --- |
| RASis | 39 |
| [Irbesartan](javascript:;) | 13 |
| Valsartan | 13 |
| Losartan | 6 |
| Telmisartan | 4 |
| Perindopril | 2 |
| Captopril | 1 |
| CCBs |  |
| Amlodipine | 37 |
| Nifedipine | 27 |
| Felodipine | 3 |
| Nitrendipine | 1 |
| Varapamil | 1 |
| Diuretics |  |
| Hydrochlorothiazide | 6 |
| Indapamide | 1 |
| β-blockers |  |
| Metoprolol | 16 |
| Bisoprolol | 4 |

Abbreviations: RASi, renin-angiotensin system inhibitor; CCB, calcium channel blocker.

**Supplementary Table 2. Characteristics of the study cohort before PS matching**

|  | **All**  **(n=102)** | **RASis users**  **(n=39)** | **RASis non-users**  **(n=63)** | **ASD*** |
| --- | --- | --- | --- | --- |
| Sex, Male, n (%) | 58 (56.9%) | 25 (64.1%) | 33 (52.4%) | 0.241 |
| Age (years) | 66.0 (58.0-72.0) | 66.0 (56.5-71.5) | 66.0 (59.0-72.0) | -0.098 |
| Anti-hypertensive drugs, n (%) |  |  |  |  |
| CCBs | 69 (67.6%) | 27 (69.2%) | 42 (66.7%) | -0.055 |
| Diuretics | 7 (6.9%) | 6 (15.4%) | 1 (1.6%) | 0.377 |
| β-blockers | 20 (19.6%) | 11 (28.2%) | 9 (14.3%) | 0.305 |
| Blood pressure |  |  |  |  |
| SBP (mmHg) | 138 (127-146) | 139 (124-147) | 137 (128-146) | -0.171 |
| DBP (mmHg) | 81 (74-90) | 78 (72-89) | 82 (76-91) | -0.215 |
| Diagnosis, n (%) |  |  |  |  |
| iCCA | 68 (66.7%) | 23 (59.0%) | 45 (71.4%) | -0.250 |
| pCCA | 24 (23.5%) | 9 (23.1%) | 15 (23.8%) | -0.017 |
| dCCA | 10 (9.8%) | 7 (17.9%) | 3 (4.8%) | 0.339 |
| Tumor size (mm) | 45.0 (22.0-64.0) | 43.0 (20.5-57.5) | 50.0 (29.0-64.0) | 0.210 |
| Perineural invasion, n (%) | 37 (36.3%) | 15 (38.5%) | 22 (34.9%) | 0.072 |
| Vascular cancer embolus, n (%) | 10 (9.8%) | 4 (10.3%) | 6 (9.5%) | 0.024 |
| Adjacent organ invasion, n (%) | 5 (4.9%) | 3 (7.7%) | 2 (3.2%) | 0.167 |
| Intrahepatic metastasis, n (%) | 8 (7.8%) | 3 (7.7%) | 5 (7.9%) | -0.009 |
| Lymph node metastasis, n (%) | 25 (24.5%) | 7 (17.9%) | 18 (28.6%) | -0.273 |
| Biliary stone, n (%) | 35 (34.3%) | 14 (35.9%) | 21 (33.3%) | 0.053 |
| TBIL (μmol/L) | 17.4 (11.7-67.6) | 17.3 (12.9-67.0) | 17.4 (10.9-84.8) | -0.031 |
| DBIL (μmol/L) | 3.7 (2.2-56.0) | 3.5 (2.1-70.3) | 3.7 (2.7-31.5) | -0.184 |
| IBIL (μmol/L) | 13.3 (9.3-42.7) | 13.3 (8.9-44.6) | 13.2 (10.6-38.6) | 0.014 |
| ALB (g/L) | 38.9 (36.2-41.4) | 38.3 (36.3-40.5) | 39.7 (36.2-41.8) | -0.171 |
| ALBI score | -2.47 (-2.73--1.97) | -2.43 (-2.60--2.06) | -2.55 (-2.77--1.95) | 0.036 |
| CA19-9 (U/L) | 124.8 (18.1-1116.9) | 216.0 (23.2-958.4) | 81.3 (17.0-1197.5) | 0.059 |
| CEA (ng/ml) | 3.0 (2.1-6.3) | 3.1 (2.2-6.1) | 2.8 (2.0-6.1) | 0.019 |
| CA125 (U/L) | 17.7 (10.7-35.8) | 16.9 (10.8-33.7) | 20.0 (11.5-40.0) | 0.042 |
| Adjuvant therapy, n (%) | 22 (21.6%) | 6 (15.4%) | 16 (25.4%) | -0.274 |

Continuous variables were expressed as median with interquartile range.

Categorical variables were expressed as number (%).

Abbreviations: PS, propensity score; ASD, absolute standardized difference; RASi, renin-angiotensin system inhibitor; CCB, calcium channel blocker; SBP, systolic blood pressure; DBP, diastolic blood pressure; iCCA, intrahepatic cholangiocarcinoma; pCCA, perihilar cholangiocarcinoma, dCCA, distal cholangiocarcinoma; TBIL, total bilirubin; DBIL, direct bilirubin; IBIL, indirect bilirubin; ALB, albumin; ALBI, albumin-bilirubin; CA19-9, carbohydrate antigen 19-9; CEA, carcinoembryonic antigen; CA125, cancer antigen 125.

* Variables with an ASD >0.20 is considered to be not well balanced.

**Supplementary Table 3. Supplementary characteristics of patients in the whole study cohort**

| **Characteristics** | **All**  **(n=102)** | **RASis users**  **(n=39)** | **RASis non-users**  **(n=63)** | ***P* value** |
| --- | --- | --- | --- | --- |
| UREA (mmol/L) | 5.0 (4.2-6.5) | 5.7 (4.6-7.3) | 4.7 (4.0-5.7) | 0.001 |
| CREA (μmol/L) | 72 (59-82) | 76 (68-88) | 69 (54-79) | 0.067 |
| K^+^ (mmol/L) | 3.98 (3.71-4.29) | 4.02 (3.72-4.38) | 3.96 (3.64-4.21) | 0.340 |

Variables were expressed as median with interquartile range.
CREA, creatinine; K+, serum potassium.

**Supplementary Table 4. Supplementary characteristics of patients in the cohort after PS matching**

| **Characteristics** | **All**  **(n=84)** | **RASis users**  **(n=28)** | **RASis non-users**  **(n=56)** | ***P* value** |
| --- | --- | --- | --- | --- |
| UREA (mmol/L) | 4.85 (4.1-6.0) | 5.4 (4.4-6.9) | 4.7 (3.9-5.7) | 0.015 |
| CREA (μmol/L) | 70 (57-80.5) | 74.5 (68-85) | 66.5 (54.5-76) | 0.017 |
| K^+^ (mmol/L) | 3.96 (3.65-4.26) | 3.95 (3.71-4.39) | 3.96 (3.64-4.13) | 0.516 |

Variables were expressed as median with interquartile range.
CREA, creatinine; K+, serum potassium.

**Supplementary Table 5. The proportion of azotemia and hyperkalemia of the whole study cohort**

| **Characteristics** | **All**  **(n=102)** | **RASis users**  **(n=39)** | **RASis non-users**  **(n=63)** | ***P* value** |
| --- | --- | --- | --- | --- |
| UREA, >8.6mmol/L | 7 (6.9%) | 5 (12.8%) | 2 (3.2%) | 0.142 |
| CREA, >115μmol/L | 7 (6.9%) | 5 (12.8%) | 2 (3.2%) | 0.142 |
| K^+^,>5.5mmol/L | 1 (1.0%) | 0 (0.0%) | 1 (1.6%) | 1.000 |

Variables were expressed as number (%).
CREA, creatinine; K+, serum potassium.

**Supplementary Table 6. The proportion of azotemia and hyperkalemia of the cohort after PS matching**

| **Characteristics** | **All**  **(n=84)** | **RASis users**  **(n=28)** | **RASis non-users**  **(n=56)** | ***P* value** |
| --- | --- | --- | --- | --- |
| UREA, >8.6mmol/L | 3 (3.6%) | 2 (7.1%) | 1 (1.8%) | 0.533 |
| CREA, >115μmol/L | 3 (3.6%) | 2 (7.1%) | 1 (1.8%) | 0.533 |
| K^+^,>5.5mmol/L | 1 (1.2%) | 0 (0.0%) | 1 (1.8%) | 1.000 |

Variables were expressed as number (%).
CREA, creatinine; K+, serum potassium.
